# Adipose Dysfunction Indices as a Key to Cardiometabolic Risk Assessment—A Population-Based Study of Post-Myocardial Infarction Patients

**DOI:** 10.3390/metabo14060299

**Published:** 2024-05-24

**Authors:** Elżbieta Szczepańska, Małgorzata Słoma-Krześlak, Agnieszka Białek-Dratwa, Izabela Dudzik, Oskar Kowalski

**Affiliations:** 1 Department of Human Nutrition, Department of Dietetics, Faculty of Public Health in Bytom, Medical University of Silesia in Katowice, ul. Jordana 19, 41-808 Zabrze, Poland; 2 Department of Cardiology, Congenital Heart Diseases and Electrotherapy, Silesian Center for Heart Diseases, ul. Marii Curie-Skłodowskiej 9, 41-800 Zabrze, Poland

**Keywords:** adipose dysfunction, cardiometabolic risk, adipose, myocardial infarction, body composition

## Abstract

Anthropometric indices, such as the BMI (body mass index), WC (waist circumference), and WHR (waist–hip ratio) are commonly used for cardiometabolic risk assessment. Consequently, in the context of evaluating cardiometabolic risk in the post-MI population, it is worthwhile to consider indices such as the Visceral Adiposity Index (VAI) and Body Adiposity Index (BAI), which have emerged as valuable risk assessment tools in clinical trials. The aim of this study was to provide a more comprehensive understanding of the importance of anthropometric indices and body composition analysis in evaluating the cardiometabolic risk among post-myocardial infarction patients. In the pursuit of this objective, this study involved assessing the BMI, WC, WHR, WHtR, VAI, BAI, and body composition in a population of patients. This study enrolled a total of 120 patients hospitalised at the Silesian Centre for Heart Diseases (SCCS) due to MI, and body composition analysis evaluated various parameters including the percentage of adipose tissue (FatP) [%], total adipose tissue (FatM) [kg], fat-free mass (FFM) [kg], muscle mass (PMM) [kg], total body water (TBW) [kg], and visceral adipose tissue (VFAT). The mean BMI for the entire group was 27.76 ± 4.08, with women exhibiting a significantly lower value compared with men (26.66 ± 3.33 vs. 28.16 ± 4.27). The mean values obtained for the WHR, WHtR, BAI, and VAI were 0.97 ± 0.08, 0.59 ± 0.07, 28.37 ± 5.03, and 3.08 ± 3.50, respectively. Based on the visceral adiposity index (VAI), in 47.5% patients, there was no adipose tissue dysfunction, with a higher proportion among women (71.88%) compared with men (38.64%). What raises concern is that 32.50% of patients had acute ATD, with a significantly higher prevalence among men (38.64%) compared with women (15.63%). Conclusion: The study results suggest that the BMI, WC, and WHR have their limitations, whereas the WHtR, VAI, and BAI provide a more comprehensive view of cardiometabolic risk, especially in the context of adipose tissue distribution and its metabolic consequences. Incorporating the WHtR, VAI, and BAI into routine clinical practice may enhance the management of cardiometabolic risk, especially among post-MI patients.

## 1. Introduction

Given the current global rise in the incidence of cardiovascular diseases (CVDs), evaluating cardiometabolic risk takes on a particular importance. Despite significant advancements in prevention and treatment, a high incidence of complications and recurrent cardiac events continues to be observed in the population of patients after myocardial infarction (MI) [1,2,3,4]. Consequently, understanding the significance of anthropometric indices and body composition analysis in the evaluation of cardiometabolic risk among these patients emerges as a pivotal concern.

Anthropometric indices, such as the BMI (body mass index), WC (waist circumference), and WHR (waist–hip ratio) are commonly used for cardiometabolic risk assessment. However, as indicated by some studies, these indices have only moderate predictive value for cardiovascular disease (CVD) and cardiometabolic dysregulation (CD) risk [5,6]. Anthropometric indices do not properly take into account age and gender variation. While they do indicate excess body weight, they fail to reflect the content and/or distribution of adipose tissue in the body, which are crucial aspects in clinical practice. Numerous studies have demonstrated a clear association between adipose tissue accumulation and metabolic risk or CVD risk [7,8,9].

Consequently, in the context of evaluating cardiometabolic risk in the post-MI population, it is worthwhile considering indices such as the Visceral Adiposity Index (VAI) and Body Adiposity Index (BAI), which have emerged as valuable risk assessment tools in clinical trials. From this perspective, evaluating body composition is also important. The VAI is acknowledged as a reliable indicator of adipose tissue dysfunction and CD risk [10,11]. Research indicates that the VAI is independently linked to coronary artery disease (CAD) and cerebrovascular events, such as stroke. This holds particular significance because the VAI takes into account both physical and metabolic parameters, offering an indirect insight into other non-classical risk factors. These may encompass dysregulated secretion of adipokines, increased lipolysis, and elevated plasma levels of free fatty acids. In addition, the VAI exhibits a significant correlation with all components of the metabolic syndrome and cardiovascular and cerebrovascular events [12,13,14]. The BAI, or Body Adiposity Index, is increasingly employed to predict cardiometabolic risk. It enables an accurate reflection of the change in body fat following weight loss. In summary, the VAI and BAI are crucial tools for evaluating cardiometabolic risk, particularly in the context of adipose tissue dysfunction and its role in the development of CVDs. The application of both indices in clinical practice may contribute to a better understanding and management of cardiometabolic risk, particularly in post-MI patients [15,16,17].

In addition to anthropometric indices, an important role in evaluating cardiometabolic risk is attributed to biochemical markers [18,19]. They help assess morphotic blood elements, glucose levels, and cholesterol (total and fractions), and they contribute to the evaluation of inflammation [20,21,22]. Abnormalities in blood lipid levels (dyslipidaemia) represent one of the primary risk factors for cardiometabolic disease and atherosclerosis. The lipid profile and triglyceride levels play a crucial role in risk assessment, particularly in post-MI patients, and optimizing the lipid profile through therapy represents a key element in secondary prevention [23,24].

The aim of this study was to provide a more comprehensive understanding of the importance of anthropometric indices and body composition analysis in evaluating the cardiometabolic risk among post-myocardial infarction patients. In the pursuit of this objective, this study involved assessing the BMI, WC, WHR, WHtR, VAI, BAI, and body composition in a population of patients. Subsequently, the values of the conventional indices were compared with the contemporary ones, taking into account the distribution of adipose tissue and gender-based variation. This study further analysed the correlations between the anthropometric and biochemical indices, encompassing the lipid profile and inflammatory markers. In addition, the authors evaluated gender-specific differences in the results obtained for women and men.

## 2. Material and Methodology

### 2.1. Study Group Selection

This study enrolled a total of 120 patients hospitalised at the Silesian Centre for Heart Diseases (SCCS) in Zabrze (Poland) between March 2022 and July 2023 due to MI, who met the study inclusion and exclusion criteria. The mean age of the patients was 59.79 ± 10.79 years (women: 60.56 ± 9.04 years; men: 59.51 ± 11.39 years). This study was conducted in accordance with the principles outlined in the Declaration of Helsinki. The study protocol was approved by the Ethics Committee at the Medical University of Silesia in Katowice, Poland (Resolution No. PCN/CBN/0022/KB1/91/21 of 6 July 2021). Eligible patients were provided with information about the study’s procedures and gave their informed consent to participate. The inclusion criteria for this study included age at least 18 years, hospitalisation due to recent MI (ICD-10 diagnosis code: I25.1), appropriate functional status and motor performance as determinants of independent mobility and self-care, and informed consent to participate in this study. The exclusion criteria for this study included complicated MI, conditions hindering independent mobility and self-care, implanted pacemaker or defibrillator, metal implants in the body (such as endoprostheses), history of epilepsy (excluding individuals from body composition analysis), psychosocial limitations impairing the patient’s ability to answer questions, and lack of consent to participate in this study.

### 2.2. Body Composition Analysis

This study employed Bioelectrical Impedance Analysis (BIA) for data collection and examination. For every patient, impedance measurements of the individual body segments (arms, legs, and torso) were performed with electric currents at different frequencies (segmental multifrequency BIA: 5 kHz/50 kHz/250 kHz), with an octapolar (eight-point) contact electrode system. For the measurements, the subjects assumed a standing position, with bare feet and hands. In line with the body composition testing protocol, the patients underwent measurements in the morning, while in a fasting state. Because of hospitalisation, the subjects refrained from consuming alcohol and caffeine, and engaged only in limited physical activity on the day preceding the measurement.

The tests were performed using TANITA MC-780 S MA body composition analyser. The device is approved for medical applications and complies with NAWI CLASS III standards for body scales used in medical measurements. It is EU-certified (CE 0122), and adheres to the requirements laid down in the Medical Device Directive (MDD 93/42/EEC) [25].

Body composition analysis evaluated various parameters including the percentage of adipose tissue (FatP) [%], total adipose tissue (FatM) [kg], fat-free mass (FFM) [kg], muscle mass (PMM) [kg], total body water (TBW) [kg], and visceral adipose tissue (VFAT) [with each assigned a value in points].

In line with the recommendations from the analyser’s manufacturer, total-body adipose tissue was used for determining whether the subjects’ body weight was normal. To this end, body weight standards were employed, with due consideration given to the age and gender of the patients (Table 1).

This study also assessed the amount of visceral adipose tissue (VFAT). The standards specified by the manufacturer of the body composition analyser were employed, where scores ranging from 1 to 12 represent a healthy level of visceral adipose tissue, while scores from 13 to 59 indicate excessive visceral adiposity [27].

### 2.3. Anthropometric Measurements

Body height was measured with a stadiometer to the nearest 1 cm. Hip circumference (HC) and waist circumference (WC) were also measured to the nearest 1 cm. The waist–hip-ratio (WHR) was calculated by dividing the subject’s WC by the HC. The waist-to-height ratio (WHtR) was calculated as the WC divided by height.

Using the data obtained, the Body Mass Index (BMI) was calculated. The BMI is the most widely used tool for assessing whether body weight falls within a healthy or unhealthy range [6,28]. However, since the BMI does not take into account the content and distribution of adipose tissue, the Visceral Adiposity Index (VAI) was applied for subsequent analysis. The VAI is calculated on the basis of anthropometric parameters (BMI and WC) along with biochemical markers (high-density lipoprotein (HDL) cholesterol and triglyceride levels). Then, the calculated values were interpreted separately for patients of each gender. In the next step, the Body Adiposity Index (BAI) was calculated from hip circumference and height. Existing studies have shown that the BAI can be a predictor of the percentage of body adiposity measured by DXA [29] (Table 2).

### 2.4. Statistical Analysis

Statistical analysis was carried out using Statistica v. 13.3 software from StatSoft Inc., Tulsa, OK, USA. The results are presented as means and standard deviation (X ± SD). The variables were examined using statistical tests to draw statistical conclusions. The distribution of each parameter was examined using the Shapiro–Wilk test. Thus, non-parametric tests were used for statistical analysis. The Chi2 NW test was used to test nonparametric data. Cramer’s V coefficient (Vcr) was used to determine the strength of the relationship. The V Cramer coefficient takes values from 0 to +1 (inclusive), where the closer the score is to 0, the weaker the relationship between the studied characteristics, and the closer it is to 1, the stronger the relationship between the studied characteristics. When comparing continuous variables in the two groups of subjects (men and women), the distribution was not normal and we used the Mann–Whitney U test. The Kruskal–Wallis test was used to evaluate and compare multiple independent groups. A statistical significance level of *p* < 0.05 was used for all calculations.

## 3. Results

This study enrolled a total of 120 patients hospitalised at the Silesian Centre for Heart Diseases (SCCS) in Zabrze (Poland) between March 2022 and July 2023 due to MI, who met the study inclusion and exclusion criteria. The mean age of the patients was 59.79 ± 10.79 years (women: 60.56 ± 9.04 years; men: 59.51 ± 11.39 years). Patient characteristics, encompassing anthropometric measurements, body composition, and biochemical parameters for the entire group, as well as segmented by gender, are detailed in Table 3 and Table 4.

The mean BMI for the entire group was 27.76 ± 4.08, with women exhibiting a significantly lower value compared with men (26.66 ± 3.33 vs. 28.16 ± 4.27). The mean values obtained for the WHR, WHtR, BAI, and VAI were 0.97 ± 0.08, 0.59 ± 0.07, 28.37 ± 5.03, and 3.08 ± 3.50, respectively. In each of these instances, women exhibited significantly higher index values compared with men. For the remaining parameters analysed, no differences were found between men and women (Table 3).

The mean glucose level was 7.54 mmol/L ± 2.65, and the mean HbA1C value equalled 4.40% ± 2.93. In both instances, the parameters exhibited higher values in women (7.78 mmol/L ± 1.80 and 4.55% ± 2.72, respectively) compared with men (7.45 mmol/L ± 2.90 and 4.34% ± 3.02, respectively). Importantly, elevated fasting glucose levels, in this context, should be interpreted as a consequence of a recent MI rather than being attributed to dietary irregularities or undiagnosed diabetes. This interpretation is reinforced by normal haemoglobin glycosylation levels. The mean total cholesterol level was 5.03 mmol/L ± 1.46, with a higher mean value observed in women (5.23 mmol/L ± 1.46) compared with men (4.96 mmol/L ± 1.4). The levels of all lipid parameters, regardless of gender, exceeded the target values recommended in the current ESC (European Society of Cardiology) guidelines for post-MI patients [30]. Even with the use of pharmacotherapy, the desired values were not attained, raising concerns about the potential for complications and the likelihood of more cardiac events in the long term. The mean CRP level was 12.31 mg/L ± 29.2, and it was higher in men (12.74 mg/L ± 30.4) compared with women (11.14 mg/L ± 26.06). Elevated CRP levels were a result of MI rather than an indication of infection (Table 4).

The findings suggest that there is no association between the subjects’ nutritional status, lipid parameters, glycemia, and HbA1C. This may be attributed to the inadequacy of the BMI-based classification of nutritional status or the patients’ pharmacotherapy (hypolipidaemic agents, antidiabetic drugs) (Table 5).

Normal body weight—as determined by the BMI—was observed in 30% of patients, with a similar distribution between women (31.25%) and men (29.55%). However, differences were found in the prevalence of excess body weight between women and men, both in the case of overweight and grade I and II obesity.

Based on the Body Adiposity Index (BAI), it was revealed that while it assumed normal values in 33.33% of the study patients, which is similar to the BMI-based interpretation, there was significant variation between the percentages obtained for men and women (75% vs. 18.18%) and a clear difference compared with the BMI-based assessment. Significant variation was also noted in the prevalence of overweight and obesity between the male and female subjects. Importantly, the differences remained significant when compared with the prevalence assessed using the BMI, particularly among women.

Based on the visceral adiposity index (VAI), in 47.5% patients there was no adipose tissue dysfunction, with a higher proportion among women (71.88%) compared with men (38.64%). What raises concern is that 32.50% of patients had acute ATD, with a significantly higher prevalence among men (38.64%) compared with women (15.63%).

The results of the WHR analysis show that, despite the results based on the BMI, BAI, or VAI values reported above, up to 95.83% of patients had a higher than acceptable amount of adipose tissue in the body. These findings are reflected in the observation that 52.50% of patients (more women than men) exhibited android type obesity, while 43.33% (more men than women) displayed gynoid type obesity.

Normal body weight, as defined by the WHtR, was identified in 18.33% of the subjects. Notably, the group was exclusively male (25%). These percentages suggest that there is a higher proportion of individuals with abnormal body weight than what is indicated by the BMI and BAI values.

A normal percentage of adipose tissue was present in 46.67% of the subjects, with a significant difference between genders (62.50% women compared with 40.91% men).

A normal content of visceral adipose tissue was found in 68.33% of patients, including 56.82% of men and 100% of women. This means that according to the standards outlined in Table 6, none of the women exhibited abdominal obesity (Table 6).

Even though, according to the BMI, 30% (*n* = 36) of the patients had normal body weight, based on the BAI, only 38.89% of them had normal adiposity, according to the VAI, 52.78% had no adipose tissue dysfunction, according to the WHR, 8.33% had a normal amount of adipose tissue, while based on the WHtR, 36.11% had normal body weight. Also, regarding the FATP, despite a normal BMI, only 55.56% of the subjects had normal body weight (Table 7).

## 4. Discussion

The recognition and assessment of cardiometabolic risk in post-MI patients are crucial elements in further care management and the prevention of future cardiac events. As per the guidelines, the assessment of this risk is based on the BMI, which, despite its widespread use, has a number of limitations. Consequently, alternative indices, such as the WHtR and WHR, along with recently proposed indices, including the VAI and BAI, may provide more comprehensive insights into cardiometabolic risk, particularly in the context of adipose tissue distribution and its metabolic effects [5].

In our study, only 30% of patients were found to have a normal body weight, as determined by BMI assessment. The similarity between the results of our study and the findings reported by Szadkowska et al., in their study of 77 post-MI patients, is significant. Both studies found elevated BMI values among patients (with a mean of 27.5 ± 4.5 in Szadkowska’s study) [31]. Similarly, Herrmann et al., in a study involving a total of 3579 individuals after MI, found that approximately 25% of them had a BMI consistent with obesity (over 30.1 kg/m²) [32]. In a population-based study by Khan et al., who analysed the data of adult patients derived from 10 large US prospective cohorts, the risk of cardiovascular events was found to be elevated in overweight and obese individuals compared with those with normal BMI values. The findings of the study also suggest that as the BMI increases, the risk of cardiovascular disease also tends to rise [33]. Therefore, a risk assessment, even if based solely on the BMI, clearly indicates that abnormal body weight is associated with a higher incidence of disease.

Tewari et al., based on data from multiple cross-sectional studies, a randomised controlled trial, and prospective cohort studies, argued that the WHtR was a more reliable predictor of cardiometabolic risk compared with the BMI. This may be because it assesses the distribution of adipose tissue, a key risk factor both in CD and CVD. In contrast, the BMI was not found to be superior to the WHtR in any of the studies reviewed by the authors. The case for the practical implementation of the WHtR is additionally reinforced by the fact that the index is simple and condensed into an easy-to-remember phrase: “keep your waist to less than half your height” [34]. In the context of predicting the development of metabolic syndrome, WHtR was notably superior to the BMI and waist circumference (WC) across all age groups, demonstrating its versatility as a predictive tool for cardiometabolic risk in diverse populations [35]. Another meta-analysis, involving over 300,000 adults, demonstrated the superiority of the WHtR over the WC and BMI in various contexts. It provided important evidence pointing to the WHtR as a more reliable predictor of cardiometabolic risk, especially in the context of ethnic differences [36]. These findings suggest that the WHtR may be a more suitable tool for global cardiometabolic risk assessment, taking due account of the genetic and environmental diversity across different ethnic groups. These conclusions are consistent with the findings of our own study, in which the WHtR revealed a higher prevalence of abnormal body weight compared with the BMI, which may indicate its greater utility.

While the BMI is the clinically established indicator for assessing CVD risk linked to excess body weight, important additional insights can be gained by studying the distribution of adipose tissue in the body. Excess abdominal adiposity is linked to the production of proinflammatory cytokines and the development of leptin resistance, which contributes to chronic body inflammation and is implicated in the pathophysiology of atherosclerosis and cardiovascular disease. Research also suggests that adipose tissue distribution has an impact on the body’s systemic inflammation and metabolism. Owing to its metabolic and endocrine activity, visceral adipose tissue has the potential to affect multiple signalling pathways, which can lead to insulin resistance and the production and secretion of various pro-inflammatory cytokines [37].

Significant variation in adipose tissue distribution between men and women is evident, along with changes related to age. For example, there is an observed increase in visceral adiposity in menopausal women, which may be attributed to hormonal imbalances and could elevate the risk of cardiovascular diseases [38]. Excess visceral adipose tissue (VAT) stands as an independent risk factor for cardiometabolic diseases. It increases the risk of conditions including hypertension, prediabetes, and diabetes, as well as hypercholesterolemia and hypertriglyceridemia [13].

Carter et al., in their comparison of links between body composition and cardiovascular risk factors in multi-ethnic populations, demonstrated diverse patterns associated with adiposity, body composition, and CVD risk factors. These differences were visible despite slight variation in body composition and BMI values. The BMI and adipose tissue content exhibited a positive association with systolic blood pressure and HbA1c, but the associations with lipids were generally stronger for adipose mass [39]. Similar relationships were also observed in our study. The mean values determined for glucose, HbA1c, and total cholesterol revealed gender-based variation in risk profiles; moreover, body composition may have a significant impact on CVD risk factors, irrespective of the BMI. This observation highlights the intricate nature of the interplay between body composition, CVD risk factors, and gender.

In our study, 95.83% of patients were identified as having high adiposity based on the WHR, with a higher percentage exhibiting android obesity compared with gynoid obesity. The android obesity type was significantly more prevalent in women, while the gynoid type was more common in men.

Zhang et al., in their study of patients with heart failure (HF) after revascularisation, found the WHR to be an independent risk factor for long-term prognosis. Each 0.01 unit increase in the WHR elevated the risk of major cardiovascular events by approximately 13.4%. These findings suggest that the WHR might serve as one of the key indicators in risk assessment in patients with heart failure (HF) after revascularisation [40]. In our study, adipose tissue dysfunction, as determined by the VAI, was observed in 52.5% of patients, with 32.5% exhibiting acute dysfunction. In another study, conducted in a group of 28,764 subjects, higher VAI levels were independently associated with a higher risk of heart failure. This finding suggests that the VAI may serve as a valuable visceral obesity indicator for assessing this risk [41].

Research findings highlight the pivotal role of hyperglycaemia in shaping patient outcomes and prognosis after myocardial infarction. Hyperglycaemia on hospital admission, frequently observed in patients with acute coronary syndrome, serves as a prognostic marker of mortality and in-hospital complications [42]. Other studies have shown that the presence of hyperglycaemia in patients admitted to ICUs with acute myocardial infarction elevates the risk of mortality and complications, irrespective of whether or not the patient has diabetes [43]. Helfand et al., demonstrated that patients with elevated glucose levels upon hospital admission had the highest in-hospital mortality rates (9.9%), surpassing the rates observed in individuals diagnosed with diabetes (6.5%) and those without diagnosed diabetes (7.5%) [44]. Similarly, Lin et al., in their study enrolling 9996 patients with diabetes and confirmed CAD revealed that glycaemic control, defined as HbA1c < 7%, was linked to a decreased risk of cardiovascular events, particularly in patients with a high triglyceride-glucose (TyG) index [45]. Another study, analysing data from six prospective, population-based cohort studies involving a total of 36,180 Europeans, found that HbA1c levels were independently associated with cardiovascular mortality, overall mortality, and cardiovascular morbidity. With each 10 mmol/mol rise in HbA1c, there was an associated increase in the hazard ratio for cardiovascular mortality, overall mortality, and cardiovascular disease morbidity [46].

A positive association between the occurrence of coronary heart disease and LDL cholesterol levels has been consistently found in observational studies across various populations [47]. A meta-analysis of large cohort studies found an inverse association between the serum levels of TC, LDL-C, and HDL-C and the risk of atrial fibrillation (AF), though no significant relationship was found between the TG levels and the occurrence of AF [48]. An extensive examination of 136,905 CAD hospital admissions based on data from the “Get With The Guidelines” database revealed that the mean lipid levels upon admission were 104.9 mg/dL (LDL), 39.7 mg/dL (HDL), and 161 mg/dL (triglycerides). Only approximately 17.6% of patients had LDL levels below 70 mg/dL, and only 1.4% had the desired LDL and HDL levels (LDL < 70 mg/dL with HDL ≥ 60 mg/dL). Also, it needs to be noted that 54.6% of the patients had HDL levels below 40 mg/dL. In our study, the mean lipid levels were comparable, with slightly elevated average LDL and HDL values. These findings suggest that while LDL levels can be effectively controlled in a considerable proportion of patients, managing HDL levels continues to pose a significant challenge [49].

An elevated level of C-reactive protein (CRP) following MI serves as a significant indicator for predicting future cardiovascular events. Elevated high-sensitivity CRP (hs-CRP) values typically peak within 48 to 72 h following STEMI (ST-segment elevation myocardial infarction) and gradually decrease over the subsequent weeks, returning to reference values below 10 mg/L [50]. Our study found that the mean CRP level after MI was 12.31 ± 29.2 mg/L, which aligns with observations from other studies indicating elevated CRP values in the early post-MI period. In one study, the mean CRP level was 1.89 mg/L upon hospital admission for myocardial infarction, reaching a peak of 12.10 mg/L during hospitalisation, and subsequently decreasing to 1.24 mg/L after one month [51]. The TRIUMPH study found that among 687 patients who had the hs-CRP measured one month and six months after MI, 69.8% of those with hs-CRP levels ≥2.0 mg/dL at one month still had elevated hs-CRP levels after six months [52]. It has been suggested that hsCRP plays an important role in the development of heart failure secondary to myocardial infarction [53]. CRP elevation in post-MI patients is linked to unfavourable outcomes, including left ventricular failure, increased cardiac mortality, and the risk of wall rupture [54]. Elevated hs-CRP 30 days after MI showed a link with poorer health status in raw analyses. However, the association lost its significance after adjusting for comorbidities, which implies that hs-CRP may serve as a marker for comorbidities associated with impaired health status [52].

Recognizing and assessing cardiometabolic risk in post-MI patients is crucial for managing their treatment and prevention. Despite the widespread use of the BMI, alternative measures such as the WHtR, and particularly the VAI and BAI, may provide a more comprehensive perspective on cardiometabolic risk. Body composition, including adipose tissue distribution, has a significant impact on CVD risk factors, which shows that individualizing the approach to each patient is critical. Furthermore, this study highlights the role of hyperglycaemia and elevated CRP levels as predictors of future cardiovascular events, emphasizing their importance as key risk markers in post-MI patients.

### Study Limitations

This study has several limitations that may affect the interpretation of the results. First, there is a lack of data regarding the exact number of days prior to hospitalization of patients due to acute myocardial infarction, as well as information on any intravenous fluids administered before hospitalization. This limitation prevents an assessment of the potential impact of such treatments on bioimpedance analysis results, which could introduce errors in evaluating the body composition of the patients.

Second, our study does not include detailed information about the diets of patients (either “nothing per one person” or “water only” diets) that were maintained throughout the hospitalization period following the infarction. The absence of this data makes it challenging to fully understand how these dietary restrictions could have influenced the biochemical profile, such as levels of HDL, triglycerides, and cholesterol. This limitation is significant as diet has a profound impact on lipid metabolism and could substantially modulate biochemical test results.

## 5. Conclusions

1. The study results suggest that the BMI, WC, and WHR have their limitations, whereas the WHtR, VAI, and BAI provide a more comprehensive view of cardiometabolic risk, especially in the context of adipose tissue distribution and its metabolic consequences.
2. Incorporating the WHtR, VAI, and BAI into routine clinical practice may enhance the management of cardiometabolic risk, especially among post-MI patients.
3. There is an association between adipose tissue distribution and cardiometabolic risk, with different patterns observed in men and women, which underscores the necessity for an individualised approach in risk assessment.
4. The findings of the present study show that further research is needed to explore improved methods of cardiometabolic risk assessment that take into account both anthropometric parameters, body composition assessment, and biochemical parameters.

## Figures and Tables

**Table 1:** BMI standards adopted for the study, taking into account subject age and gender [25,26].

Women	Men
Age 40–59 years	Age 20–39 years
- ≤22.9% underweight - 23.0–33.9% normal body weight - 34.0–39.9% overweight - ≥40.0% obesity	- ≤7.9% underweight - 8.0–19.9% normal body weight - 20.0–24.9% overweight - ≥25.0% obesity
Age ≥ 60 years	Age 40–59 years
- ≤23.9% underweight - 24.0–35.9% normal body weight - 36.0–41.9% overweight - ≥42.0% obesity	- ≤10.9% underweight - 11.0–21.9% normal body weight - 22.0–27.9% overweight - ≥28.0% obesity
	Age ≥ 60 years
	- ≤12.9% underveight - 13.0–24.9% normal body weight - 25.0–29.9% overveight - ≥30.0% obesity

**Table 2:** Cut-off values for assessing visceral obesity and body obesity using BAI, VAI, WHtR, WHR, and BMI.

**BAI—Body Adiposity Index**			
**Women**		**Men**	
Age 20–39 years			
0	underweight	0	underweight
21	health	8	health
33.01	overweight	21.01	overweight
39.01	obesity	26.01	obesity
Age 40–59 years			
0	underweight	0	underweight
23	health	11	health
35.01	overweight	23.01	overweight
41.01	obesity	29.01	obesity
Age 60–79 years			
0	underweight	0	underweight
25	health	13	health
38.01	overweight	25.01	overweight
43.01	obesity	31.01	obesity
**WHtR—Waist to Height Ratio**			
**Women**		**Men**	
0	malnutrition	0	malnutrition
0.36	underweight	0.36	underweight
0.43	slightly underweight	0.44	slightly underweight
0.47	normal body weight	0.47	normal body weight
0.5	overweight	0.54	overweight
0.55	significantly overweight	0.59	significantly overweight
0.59	obesity	0.64	obesity
**WHR—Waist-hip Ratio**			
**Women**		**Men**	
<0.8	gynoid type	<1	gynoid type
≥0.8	android type	≥1	android type
**VAI—Visceral Adiposity Index**			
Age < 30			
0		no ATD *	
2.53		mild ADT	
2.59		average ADT	
2.74		acute ADT	
≥30 < 42 years			
0		no ATD	
2.24		mild ATD	
2.54		average ATD	
3.13		acute ATD	
≥42 < 52 lat			
0		no ATD	
1.93		mild ATD	
2.17		averae ATD	
2.78		acute ATD	
≥52 < 66 lat			
0		no ATD	
1.94		mild ATD	
2.32		average ATD	
3.26		acute ATD	
≥66 lat			
0		no ATD	
2.01		mild ATD	
2.42		average ATD	
3.18		acuteATD	
**BMI—Body Mass Index**			
18.5–24.9		normal body weight	
25–29.9		overweight	
30–34.9		obesity I˚	
35.0–39.9		obesity II˚	
>40.0		obesity III˚	

* ATD—adipose tissue dysfunction.

**Table 3:** Anthropometric measurements and body composition in the study group of patients after MI by gender.

	Women *n* = 32	Men *n* = 88	Total *n* = 120	U Mann–Whitney Test
Hips [cm]	104.78 ± 9.49	103.47 ± 8.64	103.67 ± 8.86	*p* = 0.00981
Waist [cm]	95.20 ± 12.30	103.27 ± 13.56	101.26 ± 13.68	*p* = 0.00275
Body weight [kg]	71.28 ± 11.34	85.54 ± 14.72	81.74 ± 15.23	*p* = 0.28073
Height [cm]	163.00 ± 6.28	174.27 ± 6.26	171.25 ± 7.99	*p* = 0.00003
BMI [Kg/m^2^]	26.66 ± 3.33	28.16 ± 4.27	27.76 ± 4.08	*p* = 0.03418
WHR	0.90 ± 0.06	1.00 ± 0.07	0.97 ± 0.08	*p* = 0.00000
WHtR	0.58 ± 0.07	0.59 ± 0.07	0.59 ± 0.07	*p* = 0.00045
BAI	30.11 ± 5.50	27.74 ± 4.73	28.37 ± 5.03	*p* = 0.00000
VAI	1.74 ± 1.17	3.57 ± 3.92	3.08 ± 3.50	*p* = 0.00000
Fat P [%]	30.35 ± 6.33	24.05 ± 6.15	25.73 ± 6.78	*p* = 0.35721
PPM [kg]	49.23 ± 5.94	64.33 ± 7.8	60.30 ± 9.96	*p* = 0.35472
TBW [%]	32.27 ± 3.02	45.48 ± 5.73	41.96 ± 7.80	*p* = 0.66785

BMI (body mass index), WC (waist circumference), WHR (waist–hip ratio), WHtR (waist to height ratio), BAI (Body Adiposity Index), VAI (Visceral Adiposity Index), FatP [%] (percentage of adipose tissue), PMM [kg]—muscle mass, TBW [%] total body water.

**Table 4:** Biochemical parameters in the study group of patients after MI by gender.

	Women *n* = 32	Men *n* = 88	Total *n* = 120	Mann–Whitney U Test
GLUCOSE [mmol/L]	7.78 ± 1.80	7.45 ± 2.90	7.54 ± 2.65	*p* = 0.45621
HBA1C [%]	4.55 ± 2.72	4.34 ± 3.02	4.40 ± 2.93	*p* = 0.74543
TOTAL CHOLESTEROL [mmol/L]	5.23 ± 1.46	4.96 ± 1.47	5.03 ±1 0.46	*p* = 0.19857
HDL [mmol/L]	1.37 ± 0.38	1.20 ± 0.33	1.25 ± 0.35	*p* = 1.00000
LDL [mmol/L]	3.07 ± 1.48	4.25 ± 10.49	3.93 ± 9.02	*p* = 0.15744
TG [mmol/L]	1.53 ± 0.70	1.73 ± 1.55	1.68 ± 1.37	*p* = 0.09757
CRP [mg/L]	11.14 ± 26.06	12.74 ± 30.40	12.31 ± 29.2	*p* = 0.57873

HbA1C glycated hemoglobin, HDL high-density lipoprotein, LDL Low-density lipoprotein, TG triglycerides, CPR—C-reactive protein.

**Table 5:** Biochemical parameters in the study group of patients after MI by BMI.

	Body Weight according to BMI				*p* Value Kruskal–Wallis
	Normal Body Weight *n* = 36	Overweigjt *n* = 53	Obesity I˚ *n* = 24	Obesity II˚ *n* = 7	
GLUCOSE [mmol/L]	7.36 ± 3.13	7.46 ± 2.48	7.90 ± 2.54	7.87 ± 1.80	*p* = 0.085
HBA1C	4.21 ± 3.10	4.80 ± 2.76	3.84 ± 3.12	4.24 ± 2.96	*p* = 0.99
TOTAL CHOLESTEROL [mmol/L]	4.61 ± 1.22	5.16 ± 1.36	5.36 ±1.91	5.04 ± 1.51	*p* = 1.00
HDL [mmol/L]	1.24 ± 0.29	1.27 ± 0.41	1.24 ±0.33	1.12 ± 0.16	*p* = 0.54
LDL [mmol/L]	2.83 ± 1.19	5.00 ± 13.49	3.40 ±1.44	3.29 ± 1.34	*p* = 0.93
TG [mmol/L]	1.47 ± 0.88	1.70 ± 1.49	1.72 ±1.47	2.39 ± 2.16	*p* = 1.00

HbA1C glycated hemoglobin, HDL high-density lipoprotein, LDL low-density lipoprotein, TG triglycerides.

**Table 6:** Anthropometric parameters in the study group of patients after MI by gender.

Anthropometric Measurement Results	Men *n* = 88		Women *n* = 32		Total *n* = 120		*p* Value Chi ^2^ NW
	n	%	n	%	n	%	
**BMI**							
normal body weight	26	29.55%	10	31.25%	36	30.00%	*p* = 0.18009
overweight	35	39.77%	18	56.25%	53	44.17%	
obesity I˚	21	23.86%	3	9.38%	24	20.00%	
obesity II˚	6	6.82%	1	3.13%	7	5.83%	
**BAI**							
underweight	0	0.00%	3	9.38%	3	2.50%	*p* = 0.00000 V Cr = 0.630
health	16	18.18%	24	75.00%	40	33.33%	
overweight	42	47.73%	4	12.50%	46	38.33%	
obesity	22	25.00%	0	0.00%	22	18.33%	
out of range	8	9.09%	1	3.13%	9	7.50%	
**VAI**							
no ATD	34	38.64%	23	71.88%	57	47.50%	*p* = 0.00237 V Cr = 0.319
mild ATD	8	9.09%	0	0.00%	8	6.67%	
average ATD	12	13.64%	4	12.50%	16	13.33%	
acute ATD	34	38.64%	5	15.63%	39	32.50%	
**WHR**							
normal body weight	5	5.68%	0	0.00%	5	4.17%	*p* = 0.00000 V Cr = 0.498
android type	33	37.50%	30	93.75%	63	52.50%	
gynoid type	50	56.82%	2	6.25%	52	43.33%	
**WHtR**							
underweight	0	0.00%	2	6.25%	2	1.67%	*p* = 0.00026 V Cr = 0.389
slightly underweight	1	1.14%	0	0.00%	1	0.83%	
normal body weight	22	25.00%	0	0.00%	22	18.33%	
overweight	26	29.55%	7	21.88%	33	27.50%	
significantly overweight	18	20.45%	9	28.13%	27	22.50%	
obesity	21	23.86%	14	43.75%	35	29.17%	
**FATP**							
underweight	3	3.41%	5	15.63%	8	6.67%	*p* = 0.00144 V Cr = 0.346
standard	36	40.91%	20	62.50%	56	46.67%	
overweight	29	32.95%	6	18.75%	35	29.17%	
obesity	20	22.73%	1	3.13%	21	17.50%	
**VFATL**							
healthy level	50	56.82%	32	100.00%	82	68.33%	*p* = 0.00001
excess level	38	43.18%	0	0.00%	38	31.67%	

BMI (body mass index), WC (waist circumference), WHR (waist–hip ratio), WHtR (waist to height ratio), BAI (Body Adiposity Index), VAI (Visceral Adiposity Index), FatP [%] (percentage of adipose tissue), VFAT (visceral adipose tissue).

**Table 7:** Anthropometric parameters in the study group of post-MI patients by BMI.

Anthropometric Measurement Results	Body Weight According to BMI								*p* Value Kruskal–Wallis
	Normal body Weight *n* = 36		Overweight *n* = 53		Obesity I˚ *n* = 24		Obesity II˚ *n* = 7		
	n	%	n	%	n	%	n	%	
**BAI**									
underweight	3	8.33%	0	0.00%	0	0.00%	0	0.00%	*p* = 0.4335
health	14	38.89%	18	33.96%	7	29.17%	1	14.29%	
overweight	11	30.56%	23	43.40%	8	33.33%	4	57.14%	
obesity	4	11.11%	8	15.09%	8	33.33%	2	28.57%	
out of range	4	11.11%	4	7.55%	1	4.17%	0	0.00%	
**VAI**									
no ATD	19	52.78%	26	49.06%	10	41.67%	2	28.57%	*p* = 0.756
mild ATD	2	5.56%	3	5.66%	2	8.33%	1	14.29%	
average ATD	4	11.11%	8	15.09%	4	16.67%	0	0.00%	
acute ATD	11	30.56%	16	30.19%	8	33.33%	4	57.14%	
**WHR**									
normal body weight	3	8.33%	2	3.77%	0	0.00%	0	0.00%	*p* = 0.867
android type	16	44.44%	28	52.83%	14	58.33%	5	71.43%	
gynoid type	17	47.22%	23	43.40%	10	41.67%	2	28.57%	
**WHtR**									
underweight	2	5.56%	0	0.00%	0	0.00%	0	0.00%	*p* = 0.072
slightly underweight	1	2.78%	0	0.00%	0	0.00%	0	0.00%	
normal body weight	13	36.11%	7	13.21%	1	4.17%	1	14.29%	
overweight	8	22.22%	18	33.96%	5	20.83%	2	28.57%	
significantly overweight	8	22.22%	8	15.09%	11	45.83%	0	0.00%	
obesity	4	11.11%	20	37.74%	7	29.17%	4	57.14%	
**FATP**									
underweight	7	19.44%	0	0.00%	1	4.17%	0	0.00%	*p* = 0.0005 V Cr = 0.317
standard	20	55.56%	28	52.83%	6	25.00%	2	28.57%	
overweight	6	16.67%	20	37.74%	8	33.33%	1	14.29%	
obesity	3	8.33%	5	9.43%	9	37.50%	4	57.14%	
**VFATL**									
healthy level	33	91.67%	38	71.70%	7	29.17%	4	57.14%	*p* = 0.0000 V Cr = 0.472
excess level	3	8.33%	15	28.30%	17	70.83%	3	42.86%	

BMI (body mass index), WC (waist circumference), WHR (waist–hip ratio) WHtR (waist to height ratio), BAI (Body Adiposity Index), VAI (Visceral Adiposity Index), FatP [%] (percentage of adipose tissue), VFAT (visceral adipose tissue).

## Data Availability

The data presented in this study are available on request from the corresponding author. The data are not publicly available due to restrictions that apply to the availability of these data.
